# Spatially inhomogeneous electron state deep in the extreme quantum limit of strontium titanate

**DOI:** 10.1038/ncomms12974

**Published:** 2016-09-29

**Authors:** Anand Bhattacharya, Brian Skinner, Guru Khalsa, Alexey V. Suslov

**Affiliations:** 1 Materials Science Division, Argonne National Laboratory, Argonne, Illinois 60439, USA; 2 Massachusetts Institute of Technology, 77 Mass Ave, Cambridge, Massachusetts 02139, USA; 3 Center for Nanoscale Science and Technology, National Institute of Standards and Technology, Gaithersburg, Maryland 20899, USA; 4 National High Magnetic Field Laboratory, 1800 E. Paul Dirac Dr, Tallahassee, Florida 32310, USA; 6 Present address: Department of Materials Science and Engineering, Cornell University, 126 Bard Hall, Ithaca, NY 14853-1501

## Abstract

When an electronic system is subjected to a sufficiently strong magnetic field that the cyclotron energy is much larger than the Fermi energy, the system enters the extreme quantum limit (EQL) and becomes susceptible to a number of instabilities. Bringing a three-dimensional electronic system deeply into the EQL can be difficult however, since it requires a small Fermi energy, large magnetic field, and low disorder. Here we present an experimental study of the EQL in lightly-doped single crystals of strontium titanate. Our experiments probe deeply into the regime where theory has long predicted an interaction-driven charge density wave or Wigner crystal state. A number of interesting features arise in the transport in this regime, including a striking re-entrant nonlinearity in the current–voltage characteristics. We discuss these features in the context of possible correlated electron states, and present an alternative picture based on magnetic-field induced puddling of electrons.

## Abstract (web summary)

At sufficiently strong magnetic fields and low temperatures, electrons assume a quasi-one-dimensional quantum state that is challenging to observe. Here, Bhattacharya *et al*. report on electron transport in lightly-doped single crystals of SrTiO_3_ deep in this extreme quantum limit.

## Graphical abstract

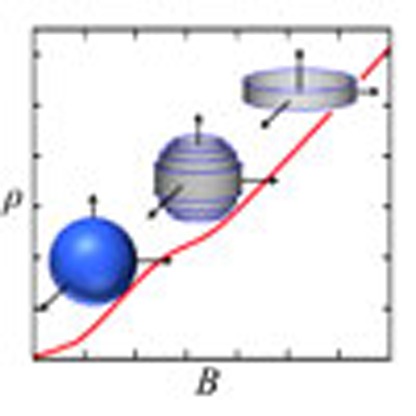

When subjected to a sufficiently strong magnetic field, the bulk properties of an electronic system change dramatically. In particular, a three-dimensional electron gas exhibits very different behaviour when the magnetic field *B* becomes large enough that the cyclotron energy *ħω*_c_=*ħeB*/*m* exceeds the Fermi energy *E*_F_∝*ħ*^2^*n*^2/3^/*m*. (Here, *ω*_c_ is the cyclotron frequency, *ħ* is the reduced Planck constant, −*e* is the electron charge, *m* is the effective electron mass and *n* is the electron density.) In this extreme quantum limit (EQL) electron motion in the directions perpendicular to the magnetic field is quantized, and all electrons occupy only the lowest Landau level. Semi-classically, one can visualize the EQL as the state in which electron trajectories are tight spirals along the field direction, with gyration radius 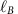 that is much shorter than the typical inter-electron spacing.

As a consequence of this quantization by magnetic field, in the EQL the electron kinetic energy retains a dependence only on the momentum parallel to the field, and the Fermi surface takes the form of two parallel rings in momentum space. For a clean, low-temperature electron gas, such a dimensionally reduced dispersion implies a number of potentially competing instabilities, including spin or valley density wave, charge density wave (CDW) and Wigner crystallization^1^,^2^,^3^,^4^.

Such a high-field situation, however, is difficult to realize experimentally. For a typical metal, for example, the EQL requires fields on the order of 10^5^ T, and is thus relevant only for extreme astrophysical settings^2^,^5^. Realization of the EQL in the laboratory requires a material that can exhibit metallic behaviour at very low electron density *n*.

Doped bulk strontium titanate, SrTiO_3_ (STO), is such a material. STO, a semiconducting perovskite oxide, has been studied intensively in recent years, with much attention devoted to its potential in thin films and heterostructure devices^6^,^7^,^8^,^9^,^10^. But as a bulk material STO has attracted interest for over half a century^11^,^12^,^13^,^14^,^15^,^16^,^17^,^18^, largely because of its anomalous dielectric response at low temperature^11^,^14^,^15^. Indeed, STO has a static, long-wavelength dielectric constant *ɛ* that reaches ≈24,000 at low temperatures (with a weak directional dependence^15^). One implication of this enormous dielectric constant is that the effective Bohr radius *a*_B_=4*πɛ*_0_*ɛħ*^2^/*me*^2^ associated with shallow donor states becomes extremely large: *a*_B_≈760 nm. Consequently, in the absence of compensating acceptors, even a very small concentration of electron donors is sufficient to ensure that STO is on the conducting side of the Mott criterion, *n*^1/3^*a*_B_≳0.2 (ref. 19). Importantly, as we discuss below, this large dielectric constant also implies a significant robustness against localization by charge disorder.

Crucial to studying the deep EQL is the existence of a strong hierarchy of energy scales:


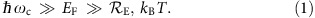


here, 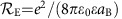 is the effective Rydberg energy and *k*_B_*T* is the thermal energy. The first inequality in equation (1) is equivalent to the large magnetic field condition explained above, while the second set of inequalities guarantees that the electron state deep in the EQL is not destroyed either by thermal excitation or by freezeout of electrons onto donor impurities. While previous high-field studies have managed to approach or even enter the EQL in doped semiconductors, simultaneously achieving both sets of inequalities in equation (1) has been more challenging. For example, studies of the EQL in narrow band gap semiconductors, such as *n*-type InAs^20^, InSb^20^,^21^,^22^,^23^ and HgCdTe^22^,^23^, are generally limited to the case where *E*_F_ and 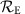 are similar in magnitude. Consequently, in these materials electrons freeze onto donor impurities shortly after the EQL is reached, and achieving a deep-EQL electron state is not possible. Previous high-field studies of STO have also generally failed to satisfy this hierarchy, either because the temperature was too high to satisfy the second inequality^24^ or because the Fermi energy was too high to satisfy the first^25^,^26^.

In this paper we present a clear experimental realization of a deep-EQL state in STO. Using transport measurements at low temperatures and high magnetic fields, we probe deeply into the EQL in a number of low-carrier-density samples. Our samples remain good bulk conductors throughout our measurement conditions, and at large magnetic fields they exhibit a strong, re-entrant nonlinearity in the resistivity. This nonlinearity is discussed in the context of possible correlated electron states, and an alternate picture is presented based on puddling of electrons in disorder potential wells.

## Results

### Shubnikov-de Haas Oscillations

In our study, we examine millimeter-sized STO single crystals (dimensions: ≈7.5 × 7.5 × 0.5 mm), obtained from CrysTec GmbH. (*Crys Tec Gmbh, Köpenicker Str. D-12555, Berlin, Germany.* *http://www.crystec.de/crystec-d.html*. This commercial product is described in this paper in order to specify adequately the experimental procedure. In no case does such identification imply recommendation or endorsement by the National Institute of Standards and Technology, nor does it imply that it is necessarily the best available for the purpose.)

To introduce a finite concentration of conduction-band electrons, the samples were heated within a vacuum chamber following the protocol described in Section IV. This heating process is known to produce oxygen vacancies within the sample volume, which act as electron donors^27^,^28^,^29^,^30^. (In principle, a single oxygen vacancy acts stoichiometrically as a double-donor, but it is generally accepted that one of the two electron states remains tightly bound to the doubly-charged oxygen ion, so that the vacancy donates only a single electron to the conduction band^31^,^32^,^33^,^34^.) The heating temperature was varied from one sample to another in order to produce samples with different doping levels. For the samples that are the focus of this study, the resulting carrier densities ranged from (7.7±1.1) × 10^15^ cm^−3^ to (1.5±0.2) × 10^18^ cm^−3^. (Here and below, all listed values of the experimental uncertainty correspond to 95% confidence intervals.)

Importantly, in addition to the oxygen vacancies, as-grown STO crystals are known to have a significant concentration of additional impurities, mostly Fe and Al, that act as deep acceptors^35^,^36^. Indeed, previous studies based on chemical analysis and secondary ion mass spectroscopy^29^ have shown these impurities to be present at the level of ≈7 × 10^17^ cm^−3^. Since this concentration is significantly larger than the measured carrier density *n*, our samples can be described as almost-completely-compensated semiconductors, for which the number of donors and acceptors are nearly identical and thus the total concentration of charged impurities *N*_i_ is much larger than *n*. This relatively large concentration of impurities is also consistent with the measured zero-field mobility, which suggests an impurity concentration on the order of 10^18^ cm^−3^ (as shown in Supplementary Note 1). The consequences of the impurity concentration *N*_i_≫*n* for transport are discussed in detail below.

**Figure 1:**
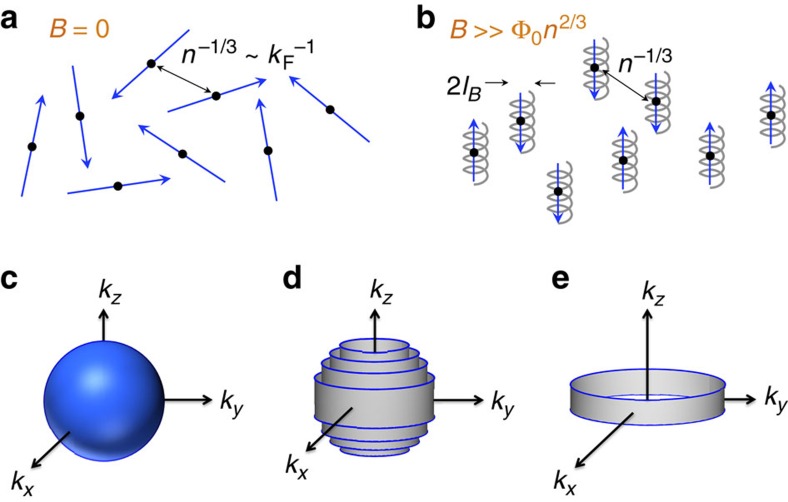
Schematic depiction of the effects of high magnetic field on a clean electron gas. (**a**,**b**) Semi-classical picture of the trajectories of Fermi level electrons, both (**a**) at zero magnetic field and (**b**) in the extreme quantum limit. *Φ*_0_ is the flux quantum and *n* is the electron density. (**c**–**e**) show the evolution of the Fermi surface (blue areas) in a clean system with increasing magnetic field. Grey areas show occupied electron states below the Fermi level. (**c**) The usual spherical Fermi surface at *B*=0. (**d**) In a strong magnetic field, electron states are quantized into Landau cylinders, each with constant magnitude of the squared transverse momentum, 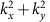. (**e**) In the extreme quantum limit, all electrons belong to the lowest Landau level.

**Figure 2:**
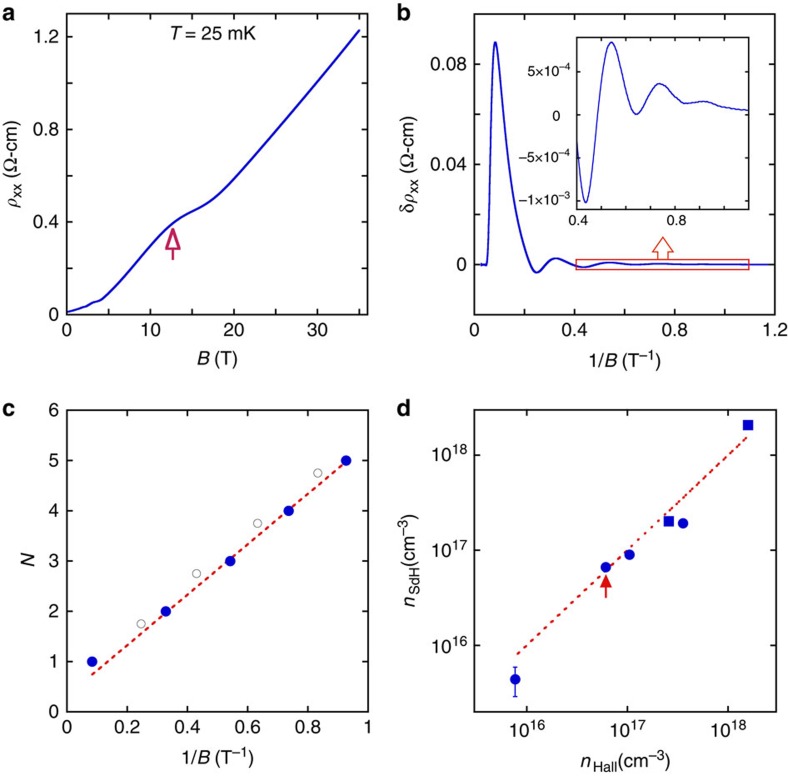
Identifying the extreme quantum limit via Shubnikov-de Haas (SdH) oscillations. (**a**) The longitudinal resistivity *ρ*_*xx*_ at *T*=25 mK for one of our samples is plotted as a function of magnetic field. The arrow indicates the onset of the EQL. (**b**) The SdH oscillations of resistivity are more easily visible if one subtracts a smooth, fourth-order polynomial from the curve in (**a**). (**c**) The index *N* is plotted against 1/*B*_*N*_. The value of *B*_*N*_ can be identified either using the local resistance maxima (filled blue circles) or the local minima of the derivative *dρ*/*dB* (open circles). Experimental uncertainty in *B*_*N*_ is ≈0.2 T, so that the uncertainty in 1/*B*_*N*_ is smaller than the symbol sizes. For the open circles, the plotted value of *N* is shifted by 1/4. (**d**) The carrier density *n*_SdH_ inferred from the SdH period (see equation (2)) is plotted against the measured Hall carrier density *n*_Hall_. Uncertainty in the value of *n*_Hall_ is about 15%, and is reflected by the size of the symbols, while uncertainty in the value of *n*_SdH_ is ≈5% except where indicated. Circles correspond to samples for which transport was measured in the 001 direction, while squares indicate measurements in the 111 direction. The arrow indicates the carrier density corresponding to the sample in parts (**a**–**c**).

As the magnetic field is increased from zero, the longitudinal resistivity exhibits Shubnikov-de Haas (SdH) oscillations, as higher Landau levels are pushed outside the Fermi surface with increasing field (as illustrated in Fig. 1). In particular, the field *B*_*N*_ at which the *N*th Landau level becomes depopulated follows^37^


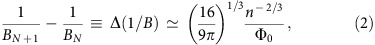


where *Φ*_0_=*πħ*/*e* is the flux quantum, and the second equality in equation (2) corresponds to the usual limit of large *N*. As shown in Fig. 2, the observed oscillations of resistivity are periodic in 1/*B*, suggesting a single, small electron pocket of Fermi surface^38^. The position of the final (*N*=1) oscillation indicates that the EQL is reached at fields *B*≈10–20 T, varying from sample to sample. Here the field *B*_*N*_ is defined experimentally as the position in magnetic field of the *N*th local maximum of resistance, counted in order of decreasing magnetic field. The identification of these maxima is facilitated by subtracting a smooth, fourth order polynomial from the *ρ* versus *B* curve, as shown in Fig. 2b (see also Supplementary Note 2 and Supplementary Fig. 1). The period of oscillation is also confirmed by the periodicity of the first derivative of *ρ* versus 1/*B* (see Fig. 2c). Plotting 1/*B*_*N*_ against the Landau index *N*, as shown in Fig. 2c, indicates unambiguously that each of our samples is well within the EQL at our largest magnetic fields.

One can note that equation (2) assumes that Landau levels are not spin-degenerate. The agreement of this equation with our measurements suggests that this is indeed the case in our samples for all appreciable magnetic fields. This non-degeneracy can be expected if one assumes that the electron *g*-factor in STO is of order unity. In this case the Zeeman energy is of order 100 *μ*eV per Tesla of field, while the Fermi energy in our samples is in the range 100 to 500 *μ*eV, so that the conduction electrons become completely spin polarized even at relatively high Landau levels.

**Figure 3:**
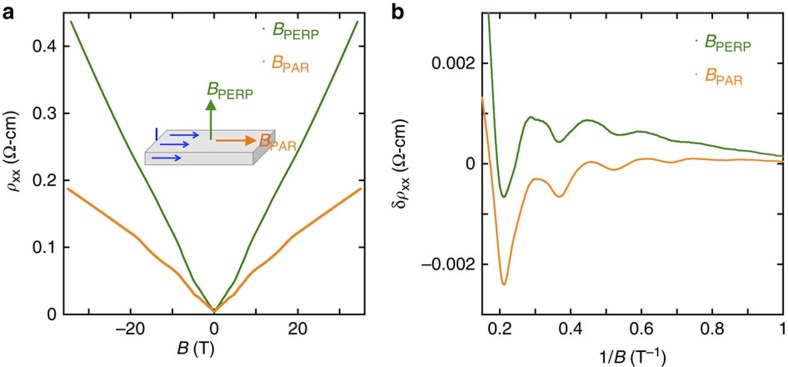
Dependence of the resistivity on field direction. (**a**) The resistivity at *T*=25 mK is shown as a function of magnetic field for both field directions. At large *B* we observe linear magnetoresistance and a significant anisotropy. (**b**) The period of the Shubnikov-de Haas oscillations is independent of whether the field is applied in the parallel or perpendicular direction.

Using equation (2), the value of the SdH period Δ(1/*B*) gives a measure of the electron density *n*. The excellent linear fit to equation (2) indicates that the large-*N* approximation is appropriate for all *N*>1. Further, the inferred value of *n* closely matches the value obtained from Hall effect measurements, as shown in Fig. 2d. (Hall measurements are presented in Supplementary Fig. 2.) The agreement between the two measures of *n* suggests that electrons are uniformly distributed through the bulk of the STO crystal, since the SdH measurements are sensitive to the average Fermi energy of electrons in the sample, while the Hall effect observes only the thickness-averaged number of carriers. The period of the SdH oscillations is also independent of whether the magnetic field is aligned parallel or perpendicular to the current, as shown in Fig. 3a, which confirms the three-dimensional nature of the electron system. We do, however, observe a noticeable anisotropy in the magnetoresistance (MR) at large fields, as shown in Fig. 3b.

Our samples also exhibit a large linear MR in the EQL, as evidenced in Figs 2a and 3b, which show a MR ratio *ρ*(*B*=45 T)/*ρ*(*B*=0)>100. Such non-saturating, linear MR is commonly associated with semi-classical drift of electron orbits along contours of a disorder potential with a long correlation length^21^,^39^,^40^, or with strong spatial inhomogeneity of the carrier concentration or mobility^41^,^42^,^43^,^44^. Below we provide an additional comment on these possibilities.

### Nonlinear transport

While the Fermi energy of a three-dimensional electron gas is essentially constant at small values of the magnetic field, once the EQL is reached (after the last SdH oscillation) the Fermi energy *E*_F_ acquires a strong dependence on the field strength. In particular, in the EQL 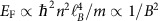, as constriction of electronic wave functions in the perpendicular directions reduces their quantum overlap and causes the Fermi energy to drop. In our samples, *E*_F_ is small enough that in the EQL only the lowest *t*_2*g*_ band is relevant^25^,^45^, and this is consistent with the single frequency observed in the low field SdH measurement. This lowest band has a slight anisotropy of the effective mass^25^, resulting from the tetragonal distortion of the STO lattice at low temperature, so for the sake of making numerical estimates below we take the effective mass *m* to be equal to the geometric mean of the three perpendicular masses, which gives *m*≈1.7*m*_0_, where *m*_0_ is the bare electron mass.

As the Fermi energy decreases with increasing field, the relative strength of the Coulomb interaction grows, as described by the ratio *α*=*E*_C_/*E*_F_, where *E*_C_∝*e*^2^*k*_F_/4*πɛ*_0_*ɛ* is the typical strength of the Coulomb interaction between neighbouring electrons in the field direction, and *E*_F_∝*ħ*^2^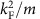 is the Fermi energy. Here, 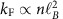 is the Fermi wave vector in the field direction, so that one can write 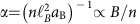. At large *α*, a clean, low-temperature electron gas becomes unstable with respect to the formation of a CDW or Wigner crystal. In our experiments *α* is as large as 12, which is two orders of magnitude larger than in previous high-field experiments in STO^25^,^26^. At such large values of *α*, Hartree-Fock calculations predict a CDW gap that exceeds the Fermi energy, indicating a strong instability toward a spatially inhomogeneous phase^46^,^47^.

**Figure 4:**
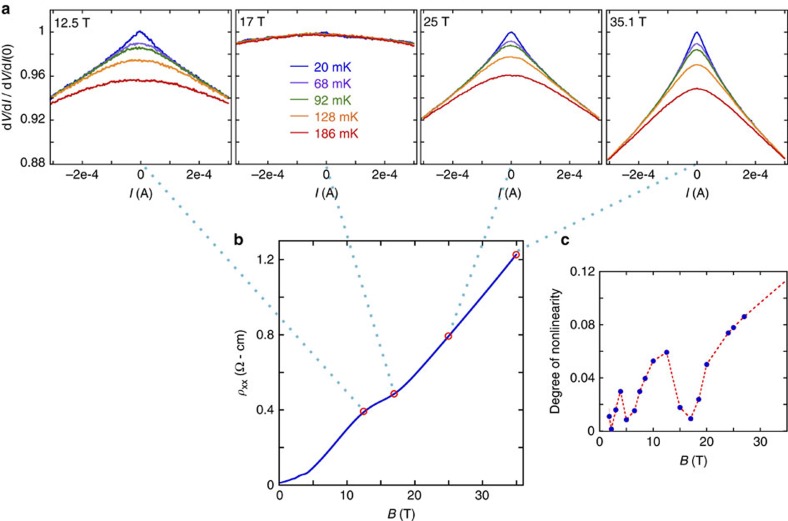
Nonlinearity of the resistance in the extreme quantum limit. (**a**) The differential resistance, d*V*/d*I*, is plotted as a function of the source-drain bias current *I* for different temperatures (different curves on each plot) and for different values of the magnetic field (different plots, each labelled by the corresponding value of *B*). For each plot, the values of d*V*/d*I* are normalized relative to the value at zero bias and *T*=20 mK. The dotted lines indicate the value of the field relative to the phase of the SdH oscillations, which are shown in (**b**). (**c**) The degree of nonlinearity in the *I*−*V* characteristics is shown to oscillate as a function of *B*, such that the transport is most nonlinear at maxima of the SdH oscillations, while at minima of the SdH oscillations the nonlinearity essentially disappears. The *y* axis corresponds to the quantity (d*V*/d*I*|_*I*=0_−d*V*/d*I*|_*I*=300 μA_)/(d*V*/d*I*|_*I*=0_) at *T*=20 mK, which approximates the slope of the top curves in **a**.

Deep within the EQL, we observe a significant nonlinearity in the current–voltage (*I*−*V*) characteristics, as illustrated in Fig. 4a. Such nonlinearity typically implies pinning or trapping of carriers by a disorder potential. The observed nonlinearity is also weaker at higher temperatures, while the zero bias resistance is lower, implying weaker pinning as temperature is increased. A careful examination of the power dissipation at low bias confirms that the nonlinearity is not a result of Joule heating, particularly at low bias (see the Methods section IV). We also find it unlikely that the nonlinearity arises from scattering by domain walls separating tetragonal domains, which form below *T*=105 K as STO undergoes a transition from cubic to tetragonal crystal symmetry^11^. The nonlinearity that we measure is clearly dependent on the magnetic field strength, while such a domain structure is *B*-independent. We also observe no sign of any anomalies in the resistivity at *T*=105 K (see Supplementary Fig. 3).

Strikingly, the observed nonlinearity appears in a re-entrant way at lower magnetic fields, becoming most pronounced at relative resistivity maxima and disappearing at relative resistivity minima. This is illustrated in Fig. 4c. It is worth noting that a similar re-entrant nonlinearity has been observed in both NbSe_3_ and InAs, although in both cases observations were limited to relatively high Landau levels. In NbSe_3_ the results were interpreted in terms of CDW physics^48^, while in InAs it was explained in terms of changes in the effective electron temperature^49^. A closer analysis of the nonlinearity is presented in Supplementary Note 3 and in Supplementary Figs 4 and 5, including the scaling of the resistivity with bias voltage and magnetic field.

## Discussion

Strong nonlinearity in the transport is expected when electrons form a spatially-correlated state, such as a CDW or Wigner crystal, that can be easily pinned by a disorder potential^50^. Such a spatially inhomogeneous state would also be consistent with the observed anisotropy in conductivity, and, indeed, previous studies of Hg_1−*x*_Cd_*x*_Te have interpreted similar nonlinearity as evidence for a Wigner crystal^51^. Working against this interpretation, however, is the very small absolute magnitude of the Coulomb interaction strength between individual electrons, owing to the large dielectric constant. Indeed, theoretical estimates of the critical temperature *T*_c_ for CDW formation^46^,^47^,^52^ suggest that *T*_c_≈5 mK or lower in our samples, and this is below our lowest measurement temperature of 20 mK. Thus, if the nonlinearity in our measurements indeed arises from a CDW or Wigner crystal phase, then it likely must be understood in combination with structural distortions in the STO lattice^53^,^54^ rather than as a simple electronic instability.

The picture of CDW-type order also ignores the role of charged impurities, which can be expected to overwhelm electron–electron interaction effects when the carrier concentration is low. An alternative picture, then, is to assume that the state of the electron system at large *B* is dictated by fluctuations in the disorder potential. In particular, if one assumes that the impurities are arranged in a spatially uncorrelated way, then random fluctuations in their local density give rise to a disorder potential with relatively large magnitude^55^,^56^


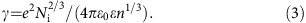


**Figure 5:**
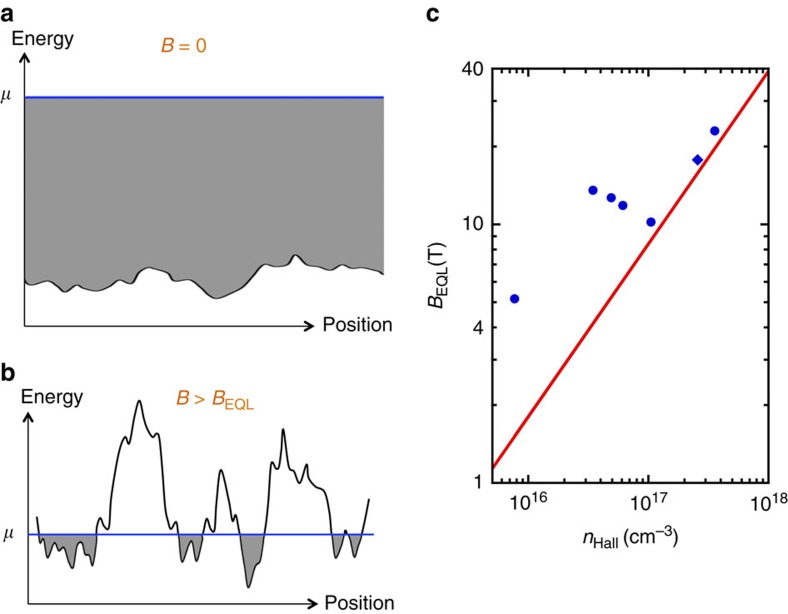
Proposed breakup of the electron liquid into puddles at large magnetic field. (**a**) At zero magnetic field, the disorder is weak compared to the typical Fermi energy, and so the electron density is nearly uniform spatially. Here, the shaded area represents filled energy levels and the blue line indicates the position of the Fermi level μ. (**b**) Deep in the extreme quantum limit, the Fermi energy is greatly reduced, and electrons reside in disconnected puddles that are localized in minima of the disorder potential. (**c**) Evidence for puddling can be seen in the relatively large value of the quantum-limiting field *B*_EQL_ for samples with low values of the electron density *n*. Symbols represent measured values of *B*_EQL_ (with the diamond symbol indicating a measurement in the 111 direction). Experimental uncertainty in *B*_EQL_ is ≈0.2 T, smaller than the symbol sizes. The solid line shows the predicted value of *B*_EQL_ for a uniform electron gas, equation (4).

In our samples, *γ* is in the range 10–20 *μ*eV. At *B*=0, this disorder represents a relatively small perturbation to the electron Fermi level, as illustrated in Fig. 5a. Deep within the EQL, however, the Fermi energy falls dramatically, and one can expect that the electron liquid breaks up into disconnected puddles that are localized in wells of the disorder potential (see Fig. 5b).

In principle, this puddled state at large *B* corresponds to an insulator, with a finite activation energy *E*_a_ for electron transport that is related to the amplitude of the disorder potential. However, owing to the large dielectric response and the relative ease of percolation between wells of the potential in three dimensions, for our samples the expected value of *E*_a_ is relatively small, *E*_a_≈0.15*γ*≈*k*_B_ × (15 mK)^56^,^57^,^58^, below our lowest measurement temperatures. In this sense the picture of electron puddles is not inconsistent with the absence of a sharp upturn in the resistivity at large fields. Clear observation of a magnetic field-driven metal-insulator transition in STO may require lower temperatures or samples with lower electron concentration. It is also possible that impurity positions have some degree of spatial correlation^59^,^60^,^61^,^62^,^63^, which would further reduce *E*_a_ by diminishing the amplitude of disorder potential fluctuations.

Support for the picture of electron puddling at large field can be found by examining the value of the field *B*_EQL_ at which the system enters the EQL. For a spinless, spatially uniform electron gas,


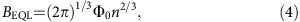


and one can therefore expect *B*_EQL_ to decrease with decreasing electron density as *B*_EQL_∝*n*^2/3^. We find, however, that *B*_EQL_ far exceeds this theoretical value for our low-density samples with *n*≲10^17^ cm^−3^, and appears to saturate at a value on the order of ≈10 T. Indeed, for our lowest-density samples, the disagreement between the observed value of *B*_EQL_ and the prediction of equation (4) is larger than 3 times, as shown in Fig. 5c. It should be emphasized that this disagreement is much larger than the inaccuracy that results from taking the large *N* limit in equation (2), which is only ≈30% when applied to *N*=1.

This discrepancy between the measured and predicted values of *B*_EQL_ can be explained within the picture of electron puddles, since the typical concentration *n*_p_ of electrons within puddles in the EQL is markedly different from the volume-averaged electron concentration *n*. Indeed, in the EQL the typical concentration of electrons within puddles takes a value that is independent of the volume-averaged electron concentration^55^,^57^:


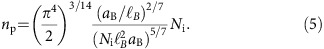


The value of *n*_p_ is determined by statistical fluctuations of the disorder potential over length scales much shorter than the correlation length of the potential. Such fluctuations are driven by the random influence of the many impurity charges, rather than by the weak nonlinear screening from the sparse electrons, and this leads to the independence of *n*_p_ on *n* implied by equation (5)^55^,^57^.

For our experiments, equation (5) suggests that *n*_p_ is on the order of a few times 10^17^ cm^−3^ in the EQL, while the typical puddle radius is ∼40 nm. One can then arrive at an estimate for *B*_EQL_ by inserting *n*_p_ from equation (5) into equation (4). This procedure gives *B*_EQL_≈10(Φ_0_/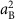)(*N*_i_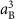)^4/9^, which for our samples is on the order of 10 T. This is consistent with the observed value in samples with small *n*≲10^17^ cm^−3^. At larger values of the doping, the carrier concentration *n* presumably becomes comparable to the total impurity concentration *N*_i_, and the electron gas becomes uniform again so that equation (4) is valid.

One can also rationalize the nonlinearity of the *I*−*V* characteristics at *B*>*B*_EQL_ within the picture of electron puddles. In particular, an applied electric field facilitates the thermal activation of electrons between adjacent puddles by adding a contribution to the potential energy that varies linearly with the electron position. This picture implies a characteristic electric field scale that is consistent with the observed nonlinearity in the EQL (see Supplementary Note 3). The nonlinearity is also identical in both the parallel and perpendicular field directions, which is again consistent with the puddle picture (Supplementary Note 3). Still, the oscillatory behaviour of the nonlinearity presented in Fig. 4 remains a prominent puzzle.

Finally, the picture of electron puddles is also qualitatively consistent with the observed linear MR in the EQL, since it corresponds to a landscape where both the disorder potential and the electron concentration have strong spatial fluctuations with a long correlation length. The observed anisotropy of resistivity is also typical in such situations^57^,^64^. (It should be noted, however, that many scenarios for electron transport in the EQL produce significant anisotropy^65^, and therefore the anisotropy by itself cannot generally be used as a strong diagnostic of the electron state.)

To summarize, this paper has presented a clear experimental demonstration of the EQL in bulk STO across a range of samples. Our experiments probe much more deeply into the EQL than previous studies of STO, and into the regime of small 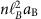, for which a charge ordering instability has long been predicted to occur for a three-dimensional electron gas. While some of our measurements are consistent with the formation of such a CDW state, including in particular a striking nonlinearity in the *I*−*V* characteristics, it seems unlikely to us that electron-electron interactions alone are sufficient to induce such a state within the regime of our experiments. Nonetheless, it remains an open question whether a field-induced CDW state can result from the combination of electron interactions and structural distortions in the STO lattice. An alternate explanation for our measurements is that the large-field behaviour is dominated by puddling of electrons in minima of the disorder potential. The observed saturation of the quantum limiting field is apparently consistent with this picture. It may be worth considering, however, whether CDW physics can coexist with an electron-puddled structure. Future studies may provide important additional insight into the electron state in the EQL, particularly if they can complement our transport measurements with thermodynamic probes like capacitance or tunnelling spectroscopy, or with studies of magnetic field-driven structural transitions.

## Methods

### Sample preparation

The SrTiO_3_ single crystals studied here were obtained from Crys Tec GmbH. On one side the samples were atomically smooth with regular unit cell high terraces, while the opposite face was unpolished. The (002) peak in all samples in this study had rocking curve full width at half maxima in the range 0.027°<Δ*ω*<0.041°. The samples were first cleaned with solvents (including trichloroethylene) to remove any residue or particulate and were then mounted on a stainless steel sample holder. All annealing was carried out using a SiC radiative heater. The samples were annealed in a prep chamber at 400 °C for ∼30 min to get rid of any adsorbed water or solvents on the holder and substrate. After the pressure of the prep chamber dropped below 4 × 10^−6^ Pa (3 × 10^−8^ torr), the sample was cooled down and inserted into the main vacuum chamber, which was typically at a base pressure between 7 × 10^−8^ Pa and 3 × 10^−7^ Pa (5 × 10^−10^ torr and 2 × 10^−9^ torr). The sample was heated to temperatures between 680 and 850 °C, depending on the doping level desired, at a rate of 30 to 40 °C min^−1^. The pressure in the chamber near the end of the annealing process was ≈3 × 10^−6^ Pa (2 × 10^−8^ torr), comprising mostly H_2_. Samples were held at the annealing temperature for exactly 60 min, after which time the power to the heater was rapidly reduced and then turned off and the sample was allowed to cool, which took ∼30 min.

### Contacts

The current contacts spanned the full width of the sample (≈0.5 × 7.5 mm), while four voltage contact tabs (with dimensions 1.5 × 1.5 mm) were deposited at the edges of the sample (spaced ≈1.8 mm apart). NiCr (50 nm)/Au (200 nm) contacts were sputtered on the unpolished side of the sample after Ar ion milling only the contact pad area for ≈15 min (400 V, 30 mA, incident at ≈45°). All samples were typically measured within a week of annealing, as the resistivity was found to increase progressively with time.

### Transport measurements

**Figure 6:**
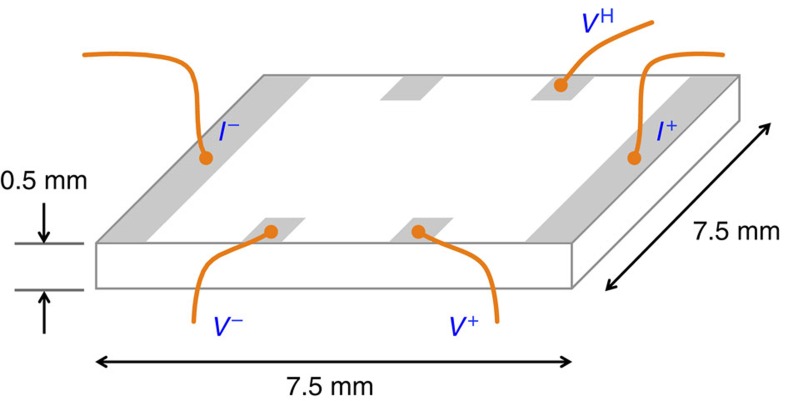
Schematic of the measurement setup. A schematic of the sample is shown, showing placement of the contacts and physical dimensions.

Simultaneous measurements of the longitudinal and Hall voltages, *V*_*xx*_ and *V*_*xy*_, respectively, were carried out in a 5-terminal Hall geometry, as illustrated in Fig. 6. Our measurements used the direct current reversal technique, and the experimental setup was an improved version of the system described in ref. 66.

Utilized current sources and nanovoltmeters have a built-in option for measurements of differential conductance or differential resistance. Thus, we used the same setup for measurements of differential resistance d*V*_*xx*_/d*I* in the 4-terminal geometry. (Elsewhere in this article, the notation d*V*/d*I* is used in place of d*V*_*xx*_/d*I*.) The differential resistance at a given bias current, *I*_bias_, was characterized by measuring changes in voltage, d*V*, associated with small excursions in the current, d*I*, about the applied bias. During the measurements *I*_bias_ was swept through a specified range with a finite step size Δ*I*_bias_.

Experiments were performed at several magnet/cryostat configurations available at the National High Magnetic Field Laboratory, and the study was performed in magnetic field as high as 45 T and at temperatures ranging from 20 to 200 mK using dilution cryostats. Care was taken to ensure that our measurements were not affected by Joule heating.

Contact resistance at low temperatures was too low to be measured reliably (1 Ω or lower). Fabrication of contacts with such low resistance was critical for carrying out low-noise voltage measurements at low temperatures, where the samples typically had resistance values in the range 0.1–100 Ω. Furthermore, for measurements in the dilution fridge, the low contact resistances at the current contacts were essential for minimizing heating due to the excitation current.

### Heating

**Figure 7:**
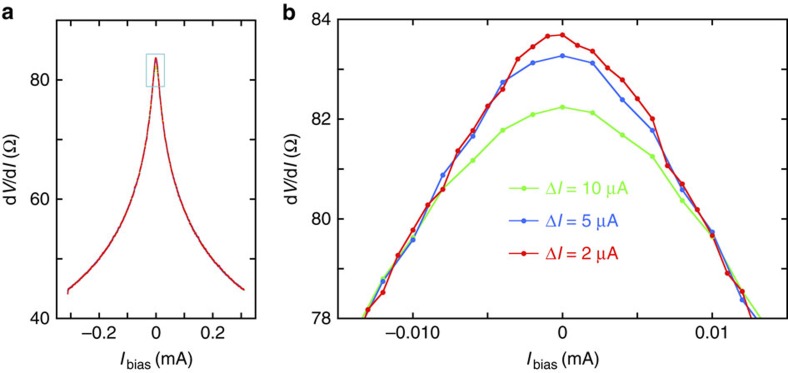
Current dependence of the differential resistance. The differential resistance, measured at temperature 20 mK and magnetic field 45 T, is shown for different values of the excitation current *I*_bias_ and the current step size Δ*I*. (**a**) Shows the measured differential resistance over a wide range of bias current. (**b**) Shows the same data plotted very near the point of zero bias. (The rectangle in (**a**) indicates the approximate range and domain of (**b**).)

To characterize the effects of the excitation current on heating in the sample, and on the value of the measured resistance in our experiments, we measured d*V*/d*I* for a range of currents between −300 and +300 μA for several values of the differential current excitation Δ*I* and the excitation current *I*_bias_. By varying these values at low *I*_bias_ we were able to substantially vary the power dissipated in a d*V*/d*I* measurement. We observed that for |*I*_bias_|>10 μA the value of d*V*/d*I* was independent of the utilized measurement parameters. This is shown for Δ*I*=2, 5 and 10 μA in Fig. 7. Our estimations show that for *I*_bias_=10 μA and Δ*I*=2 μA the power dissipated in the sample was about 57 nW, whereas for *I*_bias_=10 μA and Δ*I*=5 μA this power was about 130 nW. Thus, while corresponding values of the dissipated power differ by more than a factor of 2, the measurements nonetheless produce the same result for the differential resistance. Therefore, at least at low values of the current bias, the change in d*V*/d*I* measured as a function of bias (Fig. 4) is not due to heating. The disagreement at zero bias between the curves for Δ*I*=2, 5 and 10 μA in Fig. 7 can be explained by a coarse graining effect produced by the larger Δ*I* values near the sharp, cusp-like feature at zero bias. The data for Δ*I*=2 μA is closest to the intrinsic curve.

### Data availability

The data presented in this study are available from the corresponding authors on request.

## Additional information

**How to cite this article:** Bhattacharya, A. *et al*. Spatially inhomogeneous electron state deep in the extreme quantum limit of strontium titanate. *Nat. Commun.* **7,** 12974 doi: 10.1038/ncomms12974 (2016).

## Supplementary Material

Supplementary Information Supplementary Figures 1-5, Supplementary Notes 1-3 and Supplementary References
