# Supplementary material for: Spatially inhomogeneous electron state deep in the extreme quantum limit of strontium titanate
Source: Nat Commun. 2016 Sep 29;7:12974. doi: 10.1038/ncomms12974 (PMC5056415; doi:10.1038/ncomms12974)
Supplement: Supplementary Information — Supplementary Figures 1-5, Supplementary Notes 1-3 and Supplementary References [file ncomms12974-s1.pdf]

SUPPLEMENTARY FIGURES

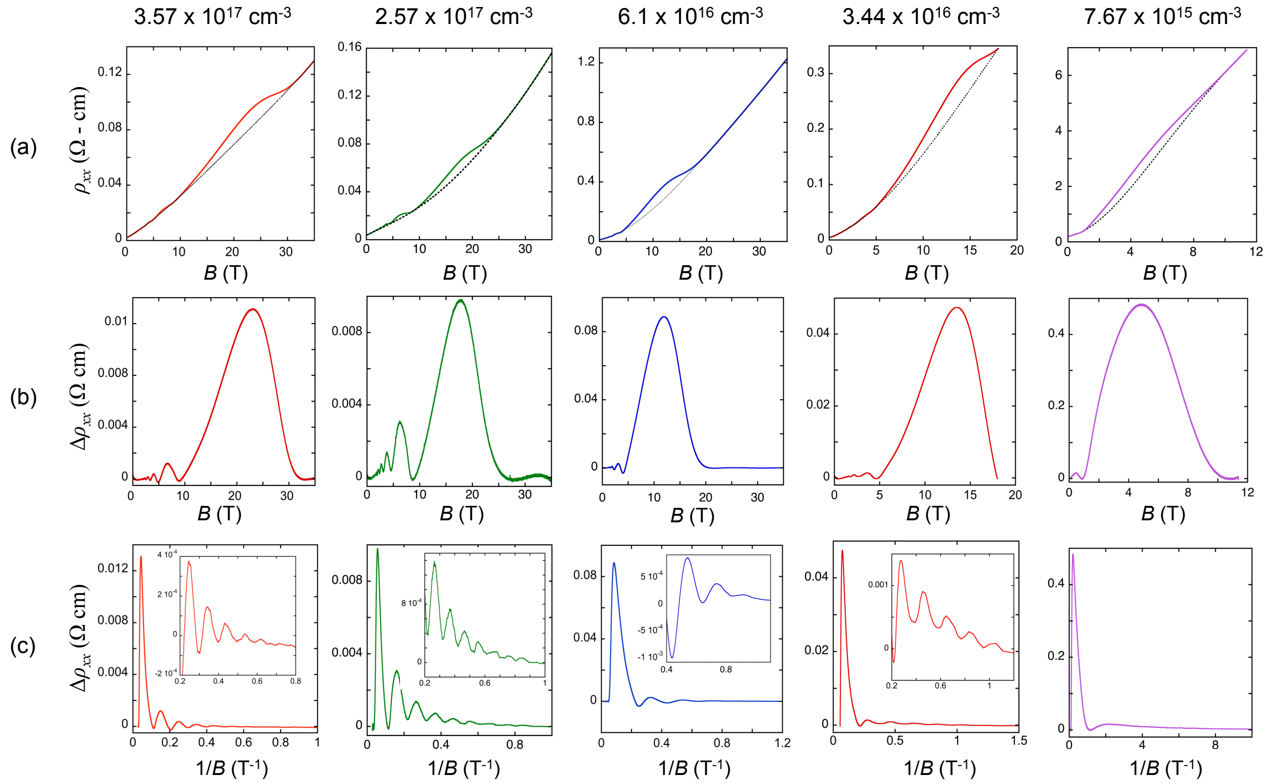

Supplementary Figure 1. Details of the Shubnikov-de Haas analysis. The longitudinal resistivity (ρ_{xx}) is plotted against magnetic field (B) at low temperatures for five of the samples studied in this work. For each sample, ρ_{xx} vs B is plotted in (a), where the dotted line depicts a fourth order polynomial which serves as a smooth background. The background subtracted data $\Delta\rho_{xx}$ are plotted vs. B in (b) and vs. $1/B$ in (c), where quantum oscillations of the resistivity are clearly visible. Insets in (c) show the same data plotted with a smaller range of the vertical axis, so that lower-amplitude oscillations are visible. Each column of plots is labeled by the corresponding value of the Hall density n_{Hall}; columns are arranged in order of decreasing n_{Hall}. All measurements were taken at $T = 25\,\mathrm{mK}$, with the exception of the sample having $n_{\mathrm{Hall}} = 3.57 \times 10^{17}\,\mathrm{cm}^{-3}$, which was measured at $T = 300\,\mathrm{mK}$.

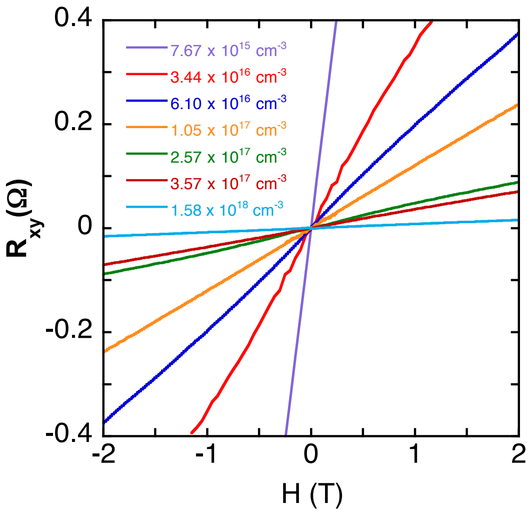

Supplementary Figure 2. Low temperature Hall data for the series of doped $SrTiO_3$ samples studied in this work. All samples are n-type, and curves are labeled by the extracted value of the Hall carrier density. All measurements were taken at $T = 25\,\mathrm{mK}$, with the exception of those corresponding to $n_{\mathrm{Hall}} = 3.57 \times 10^{17}\,\mathrm{cm}^{-3}$ and $n_{\mathrm{Hall}} = 7.67 \times 10^{15}\,\mathrm{cm}^{-3}$. The former was measured at $T = 300\,\mathrm{mK}$, and the latter was measured at $T = 2\,\mathrm{K}$.

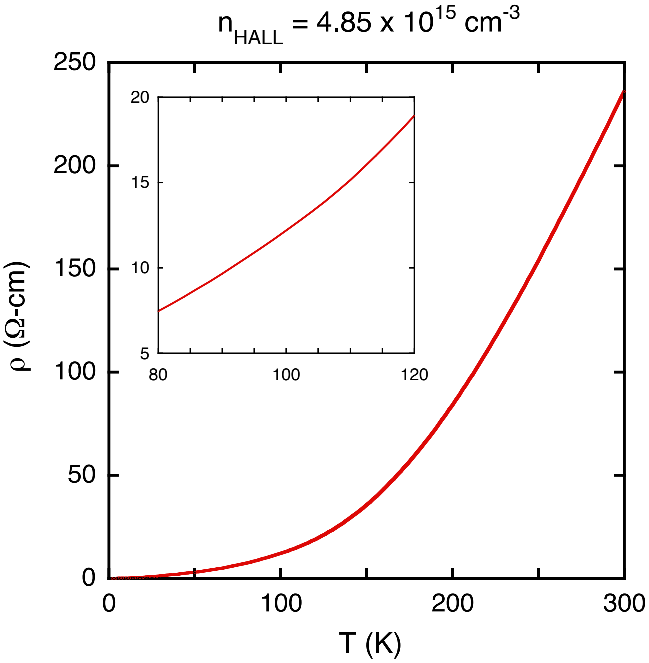

Supplementary Figure 3. Zero-field resistivity for our lowest-density sample as a function of temperature. The inset shows a detailed view of the data around $T = 105\,\mathrm{K}$. There is no sign of any anomaly in the transport at this temperature, which corresponds to a structural transition in the STO lattice.

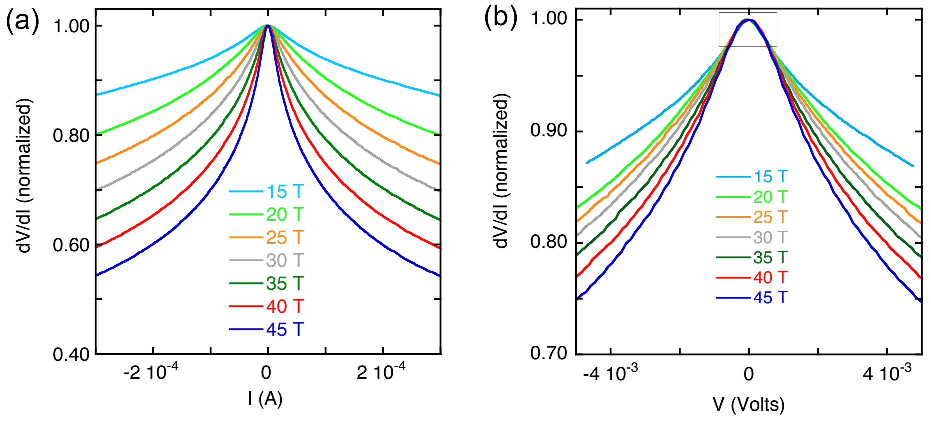

Supplementary Figure 4. Scaling of the differential resistance with bias current and bias voltage. The normalized differential resistance, $(dV/dI)/(dV/dI)|_{I=0}$, is plotted versus (a) bias current I and (b) bias voltage V at $T = 20\,\mathrm{mK}$ for a range of magnetic field values. The nonlinearity becomes stronger as the magnetic field is increased. For small values of the bias voltage V, the curve dV/dI versus V acquires a nearly universal shape, independent of the magnetic field strength.

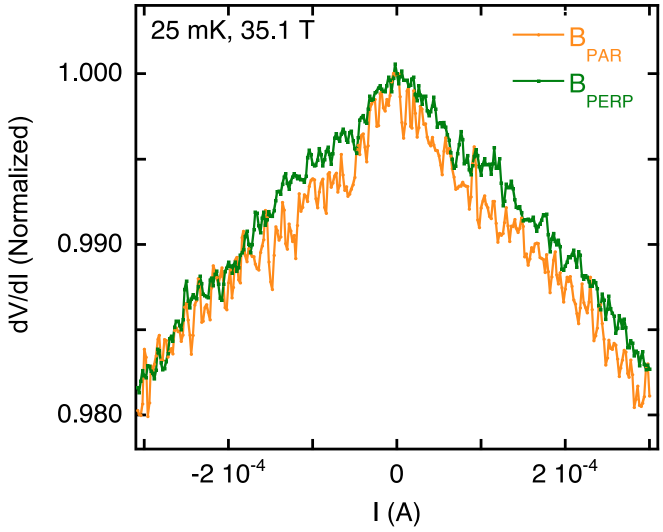

Supplementary Figure 5. Independence of the nonlinearity on the field direction. The differential resistance, dV/dI, is plotted as a function of the bias current I and normalized to the value of dV/dI at $I = 0$. The green curve corresponds to the case where the applied magnetic field is perpendicular to the bias current, while the orange curve corresponds to the case where the magnetic field and current are parallel. Measurements in this plot correspond to $T = 25\,\mathrm{mK}$ and $B = 35.1\,\mathrm{T}$.

SUPPLEMENTARY NOTE 1: ZERO-FIELD MOBILITY AND ESTIMATE OF IMPURITY CONCENTRATION

As mentioned in the main text, as-grown STO crystals generally have a relatively large number of impurities that act as deep acceptors.[1, 2] We therefore expect that the concentration of impurities N_i significantly exceeds the concentration n of free electrons. Here we show that this expectation is consistent with measurements of the zero-field, low-temperature mobility.

In particular, at low enough temperatures that the mobility saturates at a constant value, one can expect that the electron mobility μ_e is limited primarily by scattering from ionized impurities (Rayleigh scattering). For such scattering processes, screening of the impurity potential by conduction electrons is essentially irrelevant in our samples. This can be seen by examining the Thomas-Fermi screening radius

$$r_s = \sqrt{\frac{\varepsilon_0 \varepsilon}{e^2 \nu}}, \tag{1}$$

where ν is the electron density of states. In our samples $r_s \propto \sqrt{a_B / n^{1/3}} \sim 60\,\text{nm}$, which is much longer than the distance between electrons or between charged impurities. (Here, a_B denotes the effective Bohr radius at zero magnetic field.) Consequently, the screening of impurities can be ignored for calculating the scattering cross section. In this case,[3]

$$\mu_e = \frac{9\pi e n a_\text{B}^2}{2\hbar N_i \ln(3\pi^5 n a_\text{B}^3)}. \tag{2}$$

Solving Supplementary Eq. (2) for N_i gives an estimate of the impurity concentration for a given mobility μ_e and carrier density n. For the samples presented here, this estimate yields values of N_i of a few times $10^{18}\,\text{cm}^{-3}$, which is consistent with our assumptions in the main text and with previous studies.[1, 2, 4]

Of course, this value can be considered an upper-bound estimate for N_i, since the presence of other, short-ranged scatterers will also decrease the mobility. The Coulomb potential created by impurities is also not perfectly described by a constant dielectric function ε, since the dielectric function is dispersive and thus the dielectric response is not fully developed at short distances from the impurity. Hence, scattering by impurities may be somewhat stronger than implied by Supplementary Eq. (2), which would further reduce the estimate for N_i. Theoretical estimates for the dispersive nature of the dielectric function, however, suggest that this effect is not too large for isolated monovalent charges.[5]

SUPPLEMENTARY NOTE 2: SHUBNIKOV-DE HAAS ANALYSIS

In the main text we present the results of a Shubnikov-de Haas (SdH) analysis of our doped STO samples. In particular, in this analysis we identify oscillations of the longitudinal resistivity ρ_{xx} that are periodic in $1/B$. As mentioned in the main text, this is done by subtracting a smooth, fourth-order polynomial from the curve $\rho_{xx}(B)$ and identifying the field values B_N corresponding to the maxima of the background-subtracted curve $\Delta\rho_{xx}(B)$.

In Fig. 1 we show full details of this data analysis procedure for five of our studied samples, each of which is driven into the extreme quantum limit at large field. In particular, for each sample we show the raw curve $\rho_{xx}(B)$, the background-subtracted curve $\Delta\rho_{xx}(B)$, and the same data $\Delta\rho_{xx}$ plotted against the inverse field $1/B$.

SUPPLEMENTARY NOTE 3: NONLINEAR TRANSPORT

A. Resistivity Scaling

At high magnetic fields, the resistivity ρ exhibits a significant nonlinearity, such that ρ is a function of both the bias voltage V and the magnetic field B. We observe that the resistivity can in fact be scaled in such a way that different curves for the differential resistance, dV/dI, as a function of V collapse on top of each other at small V. This is shown in Fig. 4(b). The collapse of the curves suggests that one can write $dV/dI = f(V)h(B)$, where f and h are scaling functions.

From the data in Supplementary Fig. 4 one can extract a characteristic electric field scale F_0 for the nonlinearity in multiple different ways. For example, one can define F_0 as the value of the electric field above which the scaling

shown in Supplementary Fig. 4(b) is lost. This definition gives $F_0 \approx 10\,\mathrm{mV\,cm^{-1}}$. Alternatively, one could define F_0 as the value for which the differential resistance drops to half its $V = 0$ value. Such a definition gives F_0 on the order of $\approx 100\,\mathrm{mV\,cm^{-1}}$, depending on the value of B.

B. Nonlinearity in the perpendicular versus parallel field directions

Our analysis of the nonlinearity has largely focused on the case where the magnetic field direction is perpendicular to the current direction. Here we briefly show results for the case where the magnetic field and current directions are co-linear. As one can see in Supplementary Fig. 5, the two cases give essentially identical results for the nonlinear differential resistance.

C. Tetragonal domain walls

At temperatures below $T = 105\,\mathrm{K}$, STO is known to undergo a transition from cubic to tetragonal crystal symmetry.[6] Consequently, at $T < 105\,\mathrm{K}$ the sample contains domain walls between differently-oriented tetragonal domains, and these can potentially influence the electron transport. (For example, such influence has recently been studied at the STO-$LaAlO_3$ interface.[7, 8]) These domains are typically tens of microns in size, and the domain walls have a width of about 2 nm and are associated with an electronic energy scale of about 3.2 meV.[9]

In our experiments, however, we find it unlikely that these domain walls are related to the observed nonlinearity in the electron transport. Most tellingly, the features we observe in the electron transport are strongly magnetic field-dependent, while the domain structure at fixed low temperature is completely insensitive to magnetic field. In addition, we see no sign of any anomalies in the resistivity at $T = 105\,\mathrm{K}$, at which the structural transition occurs. This is shown in Supplementary Fig. 3.

D. Nonlinearity in the Electron Puddle Picture

In the picture of "electron puddles" presented in the main text, there is a natural electric field scale for the nonlinearity of the resistivity, which can be derived as follows.

For an almost-completely-compensated semiconductor in the EQL, electrons become localized in wells of the disorder potential with typical radius[10]

$$r_p \simeq (2\pi^4)^{2/7}(a_\mathrm{B}\ell_B^6)^{1/7}(N_i\ell_B^2 a_\mathrm{B})^{1/6} \tag{3}$$

and typical concentration n_p given by Eq. (4) of the main text. One can estimate the typical distance R between puddles by noting that the total number of electrons within a puddle is equal to $Q_p \simeq (4\pi/3)n_p r_p^3$, and therefore the volume-averaged concentration of electrons is $n \simeq Q_p/R^3$. Rearranging this expression for R gives a typical distance between puddles $R \simeq (4\pi n_p/3n)^{1/3} r_p$.

In the absence of a bias electric field, the typical activation energy between neighboring puddles is $E_a = c\gamma$, where γ is the typical amplitude of the disorder potential [see Eq. (2) of the main text] and c is a numerical factor that is typically ≈ 0.15.[11] One can define the typical field scale F_0 as the value of the electric field for which the difference in electric potential between puddles due to the applied field becomes equal to the activation energy E_a. In other words, $eF_0R \simeq E_a$. Rearranging this equation for F_0 and substituting $R \simeq (4\pi n_p/3n)^{1/3} r_p$ gives

$$F_0 \simeq c\left(\frac{3}{4\pi}\right)^{1/3}\frac{eN_i^{2/3}}{4\pi\varepsilon_0\varepsilon n_p^{1/3} r_p} \simeq 0.10c\frac{e}{4\pi\varepsilon_0\varepsilon}\left(\frac{N_i^3}{a_\mathrm{B}\ell_B^4}\right)^{1/7}, \tag{4}$$

where the second equality is reached by substituting Eq. (4) from the main text and Supplementary Eq. (3) into Supplementary Eq. (4).

For our samples, Supplementary Eq. (4) gives $F_0 \approx 50\,\mathrm{mV\,cm^{-1}}$ to $80\,\mathrm{mV\,cm^{-1}}$ for B between 15 T and 45 T. This range can be compared to the empirical values of F_0, which are between $10\,\mathrm{mV\,cm^{-1}}$ and $100\,\mathrm{mV\,cm^{-1}}$.

SUPPLEMENTARY REFERENCES

[1] De Souza, R. A., Metlenko, V., Park, D. & Weirich, T. E. Behavior of oxygen vacancies in single-crystal $SrTiO_3$: Equilibrium distribution and diffusion kinetics. *Physical Review B* **85**, 174109 (2012).

[2] Rice, W. D. *et al.* Persistent optically induced magnetism in oxygen-deficient strontium titanate. *Nature Materials* **13**, 481–487 (2014).
[3] Dingle, R. Scattering of electrons and holes by charged donors and acceptors in semiconductors. *Philosophical Magazine* **46**, 831–840 (1955).
[4] Spinelli, A., Torija, M. A., Liu, C., Jan, C. & Leighton, C. Electronic transport in doped $SrTiO_3$: Conduction mechanisms and potential applications. *Physical Review B* **81**, 155110 (2010).
[5] Reich, K. V., Schecter, M. & Shklovskii, B. I. Accumulation, inversion, and depletion layers in $SrTiO_3$. *Physical Review B* **91**, 115303 (2015).
[6] Cowley, R. A. Lattice Dynamics and Phase Transitions of Strontium Titanate. *Physical Review* **134**, A981–A997 (1964).
[7] Kalisky, B. *et al.* Locally enhanced conductivity due to the tetragonal domain structure in $LaAlO_3/SrTiO_3$ heterointerfaces. *Nature Materials* **12**, 1091–1095 (2013).
[8] Honig, M. *et al.* Local electrostatic imaging of striped domain order in $LaAlO_3/SrTiO_3$. *Nature Materials* **12**, 1112–1118 (2013).
[9] Cao, W. & Barsch, G. R. Landau-Ginzburg model of interphase boundaries in improper ferroelastic perovskites of d_{4h}^{18} symmetry. *Physical Review B* **41**, 4334–4348 (1990).
[10] Aronzon, B. A. & Tsidilkovskii, I. M. Magnetic-Field-Induced Localization of Electrons in Fluctuation Potential Wells of Impurities. *Physica Status Solidi (b)* **157**, 17–59 (1990).
[11] Skinner, B., Chen, T. & Shklovskii, B. I. Why Is the Bulk Resistivity of Topological Insulators So Small? *Physical Review Letters* **109**, 176801 (2012).
